# System and measurement method for binocular pupillometry to study pupil size variability

**DOI:** 10.1186/1475-925X-13-69

**Published:** 2014-06-05

**Authors:** Wioletta Nowak, Anna Żarowska, Elżbieta Szul-Pietrzak, Marta Misiuk-Hojło

**Affiliations:** 1 Institute of Biomedical Engineering and Instrumentation, Wroclaw University of Technology, Wroclaw, Poland; 2 Ophthalmology Department, Wroclaw Medical University, Wroclaw, Poland

**Keywords:** Pupillometry, Binocular measurement, Pupil size variability

## Abstract

**Background:**

An objective and noninvasive examination of pupil size variability can be used to assess the activity of the autonomous nervous system. We designed a system that enables binocular, fast, and accurate recordings of different types of pupil variabilities, which are synchronous with other biosignals. This type of measurement system is needed to extend the scope of pupillometry applications.

**Methods:**

In the proposed system, the left and right eyes are independently and interchangeably illuminated to generate alternating images, which are successively acquired by a single camera. The system is composed of four functional modules: the image acquisition module, the image processing unit, the light stimulator, and the controller. The proposed image processing algorithm approximates the shape of the pupil using the best-fit ellipse. The user control panel (controller) precisely sets the stimuli parameters and controls the entire measurement procedure.

**Results:**

The computer-based binocular system records the pupil size during the pupil light reflexes (direct and indirect) and spontaneous pupil size fluctuations, at a sampling rate up to 75 Hz, with a resolution better than 0.02 mm. Our initial laboratory tests confirmed that the new system is fast and precise (system accuracy better than 0.5% and repeatability better than 4%).

**Conclusions:**

The proposed system’s unique geometry and construction, and the method it uses to detect images from each eye, allows us to monitor the right and left eyes using a single camera with no overlap between the images. The system does not require a very experienced operator, because it is relatively simple and easy to use. Importantly, it is comfortable for the subjects. Additionally, the presented system can operate with other bio-measurement systems using a synchronous signal. These system capabilities can expand the scope of pupillometry research applications.

## Background

Pupil size variability is defined as the mobility of the pupil over time, both spontaneous and forced by external stimuli. The size of the pupil is determined by the balance of two antagonistic iris muscles: the sphincter, which reacts to parasympathetic stimuli, and the dilator, which reacts to sympathetic stimuli. Pupil size is affected by: the level of retinal illumination, the accommodation state of the eye, various sensory and emotional conditions, and cognitive and affective information processing [1].

Pupillometry refers to the measurement of pupil size and dynamics. It is a non-invasive objective monitoring technique, which is mainly used to evaluate the autonomous nervous system [2-4] and as an objective marker of light input from the retina [5].

Additionally, research has confirmed that we can use pupil response to chromatic stimuli to study in vivo a new photoreception process based on intrinsically photosensitive retinal ganglion cells [6].

Most pupillometry techniques are based on specialized video systems that record the pupil in infrared light. They can differ in their geometry, range of applications, and technical parameters. There are many commercially available pupillometers, which are often designed for a specific application. These systems can record the pupil size with a sampling frequency of 5–25 Hz for monocular registration, and of 25–60 Hz for binocular registration. The spatial resolution is in the range of 0.1–0.05 mm. There are also many laboratory solutions (prototypes) developed by different research groups, which were prepared and configured for a specific study. Monocular pupillometry systems have been previously proposed in [7,8]. Those systems used CCD linear sensors (90 Hz) and had relatively high precision parameters (0.05 and 0.01). Several monocular systems with cameras were developed in [9-11]. In those studies the eye image was recorded at a sampling rate between 62 and 155 Hz, with a spatial resolution of 0.05 mm. That research was mainly focused on the stimulus module. Another interesting monocular solution was shown in [12]. The system was based on a low cost FireWire camera. It allowed for pupil measurements at a sampling frequency of up to 120 Hz and with a spatial resolution of 0.03 mm. In [13,14], the authors proposed a video-based eye tracker. It used video cameras to record the eye position of human subjects and record pupil size and eye movements. The systems presented in [15,16] were relatively simple, and consisted of a single camera that was either attached to a slit lamp, or located at an approximately known distance from a subject positioned in a head rest. These systems recorded the pupil image at a sampling rate between 10 and 250 Hz, with a spatial resolution between 0.1 and 0.01 mm. The authors of these previous publications focused on image processing techniques that were especially devoted to pupillometry. A brief overview of pupil image analysis algorithms is presented below. In [17], the Hough transform was implemented to detect the position and diameter of a pupil modeled as a circular shape. In [18], the eye image was first resized so that the pupil is basically the only object present against the background. Then, wavelet processing was used to localize the pupil boundary, to which an ellipse was fitted. In [15], the authors proposed a fully automated procedure for pupil image segmentation based on level set theory. In [16], the pupil and iris parameters were estimated using a procedure based on the signal gradient, to localize the boundaries and fit the best ellipse. In [19], the authors proposed an algorithm that used the curvature characteristics of the pupil boundary to eliminate artefacts such as eyelids, corneal reflections, or shadows.

One of the main questions in pupillometry is whether a monocular recording is sufficient, which certainly depends on the application. If the system is intended to be used in research and clinical applications, we desire binocular registration. This is mainly for two reasons. First, the pupil light reflex is consensual. Therefore, diagnostically important information allows us to compare the direct and consensual pupil reactions of the left and right eyes. Second, pupil size may substantially vary over time as an effect of the higher centers of brain activity, which cannot be controlled. Therefore, a comparison of the size of the left and right pupils may be unreliable if they are not recorded at the same time. For example, the necessity of binocular recording is shown in the swinging flashlight test, which is used in clinical settings to detect a relative afferent pupillary defect [20].

It is also very important that the pupillometry system and measurement procedure should be easy to use, for the subject and the operator. It should not require an advanced level of cooperation from a subject, and it should not be in any way stressful or inconvenient. The calibration process should also be as short as possible and convenient. Calibrating the system to each individual subject can significantly reduce the biological noise levels, in particular with regard to eye and head movements (transversal and longitudinal). It is also important to stabilize the accommodative pupillary reflex by using a fixation point. We must also determine which type of system mounting is more practical, head or desk mounted.

In addition, it is worth noting that high-resolution parameters and comprehensive capabilities are needed if we wish to extend the scope of pupillometry research applications. A higher frame rate (although pupil activities have a relatively low frequency) means that we can: (1) determine time parameters with a higher precision, and (2) examine the time dependence of the phase between the pupil and other bio-signals. A higher spatial or linear resolution allows us to study micro-fluctuations in pupil size. High-resolution parameters mean that we can detect and quantitatively describe short time-frequency and time-amplitude variations in the pupil size using, for example, a time-frequency analysis approach [21]. The system should be able to change and adapt according to the requirements of the measurements. If we can synchronize pupil measurement with other measurements, we can extend the applications of such systems.

Our aim was to develop a measurement system to study pupil size variability that satisfies the previously mentioned factors and requirements.

## Measurement method and system

**Figure 1:**
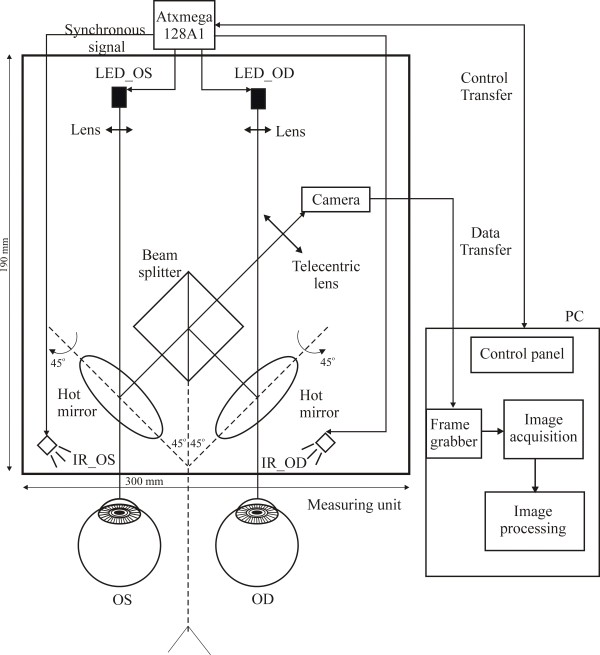
**Schematic diagram of the system.** The structure of the system and principle of its operation (the components and their arrangements relative to the eyes). Abbreviations: OS – the left eye. OD – the right eye. IR_OS – IR illuminator for the left eye. IR_OD – IR illuminator for the right eye. VIS_OS – VIS stimulator for the left eye. VIS_OD – VIS stimulator for the right eye. PC-computer.

**Figure 2:**
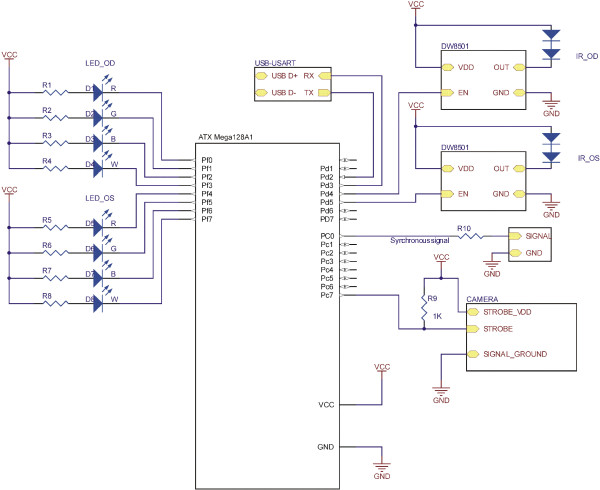
**The electrical diagram of the system.** The essential electrical components are: μP (ATX Mega 128A1), VIS diodes, IR diodes (connected with μP by DW8501 systems), connection between μP and camera, and output of the synchronous signal.

**Figure 3:**
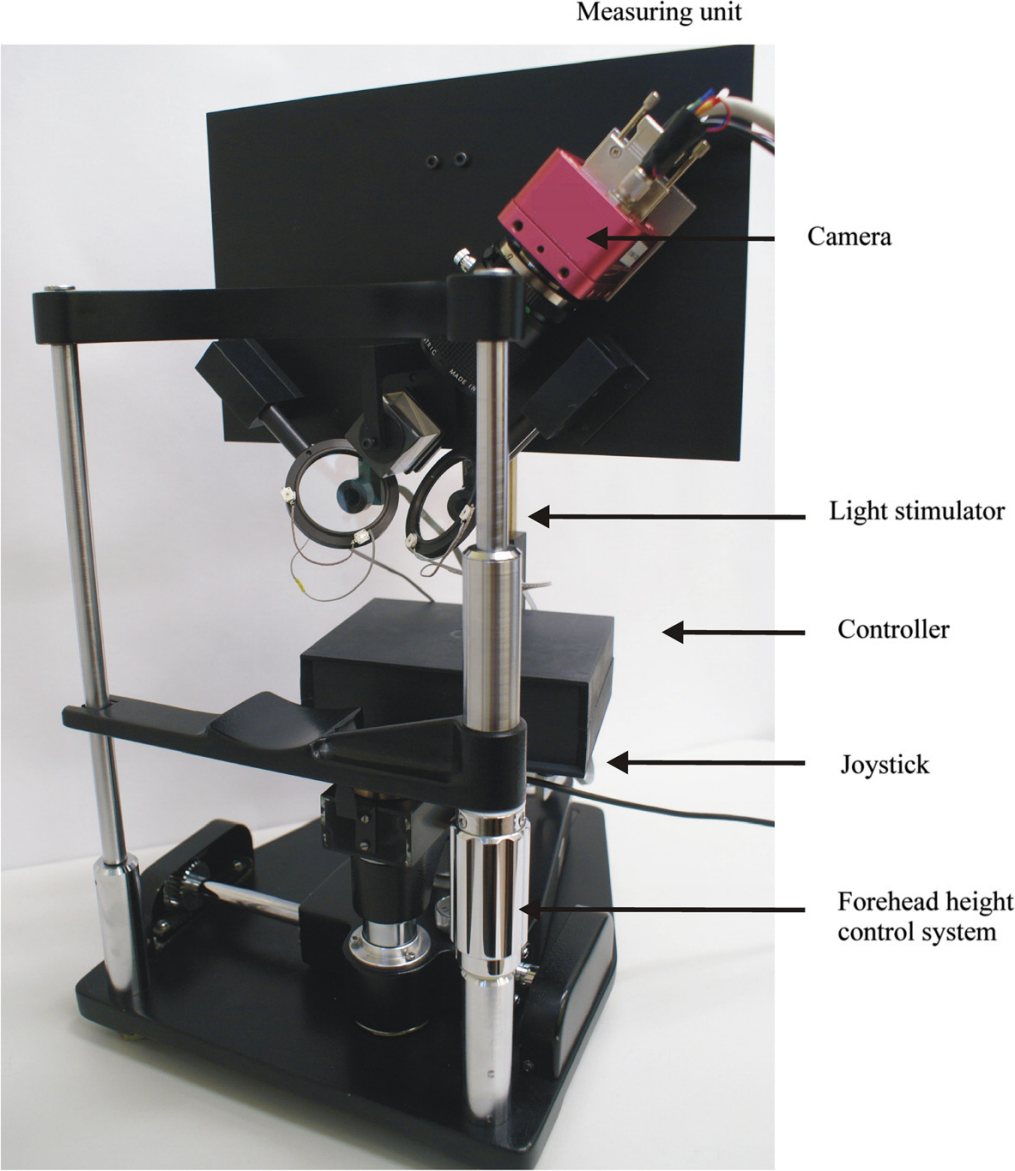
**Photograph of the system.** The measuring unit of the system is installed at the base of an ophthalmological slit lamp. In this figure, the selected system elements and forehead height control system and joystick are indicated by arrows.

In our proposed system, the left and right eyes are independently and interchangeably illuminated to generate alternating images. These images are successively transmitted by a simple optical system to the single camera with a telecentric lens. A PC stores the acquired images, and operates offline to separately calculate the pupil parameters for the left and right eyes.Figure 1 shows a schematic diagram of the system, and the principles of its operation. The electrical diagram of the system is shown in Figure 2. Figure 3 shows a photograph of the system.

The system consists of four major functional modules: the image acquisition module, the image processing unit, the light stimulator, and the controller. (The modular division is not shown in Figure 1). The components that form the measuring unit (see Figure 1) are arranged on a flat base, which is installed on an ophthalmological slit lamp base. The forehead and chin of a subject are placed against the head support. The flat base with the measuring unit is set parallel to the subject’s head. The position of the measuring unit can then be easily controlled in reference to the patient’s head. The forehead height control system and joystick can be used to adjust the system position relative to the subject during each measurement. The remaining movement systems are used to fix elements of the measuring unit, and are useful when adjusting each element during the construction stage. We discuss the construction and operation of each functional module in the following section.

## The image acquisition module

The image acquisition module consists of three parts: the Infrared (IR) illumination, the optical path, and a single camera with a frame grabber. The IR illumination part consists of four IR light-emitting diodes (LEDs) (λ = 860 nm), two for the left eye (OS) (IR_OS) and two for the right eye (OD) (IR_OD). The IR reflection from the retina allows us to take an image of the pupil in darkness, which means that the pupil size is not influenced by light. To separate the pupil and iris structures in an image, the power of total radiation is controlled and has a maximum of 10 mW, in line with the standard given in [22]. The IR light is reflected by the corneal surface, transmitted by the optical path, and projected onto a high-speed high-resolution camera. The optical path consists of two identical circular (*ϕ* = 1”) hot mirrors, a single cube beam splitter (*d* = 30 mm), and a telecentric lens with a 50-mm focal length. The diagonal of the cube beam splitter is localized parallel to the center of the subject’s nose. Mirrors are located between the cube and the eyes and are inclined at an angle of 45 degrees (with respect to the cube). The camera (Photon Focus, MV-D1024E) is a digital monochrome progressive scan camera with 1024 × 1024 pixels of size 10.6 × 10.6 μm, 150 fps at a full resolution in continuous operation, and a spectral response from 400 up to 1000 nm. The Matrix Solios type frame grabber allows the camera output to be stored in real time. The PC stores the acquired data as a sequence of gray-scale pupil images in bmp format. At full resolution, the image of each eye is recorded with a maximum sampling frequency of 75 Hz. It is worth emphasizing that the system can also record smaller images with a higher sampling rate, if required. A commercially available camera control module is used to control the image resolution and the speed of the registration.

## The image processing module

**Figure 4:**
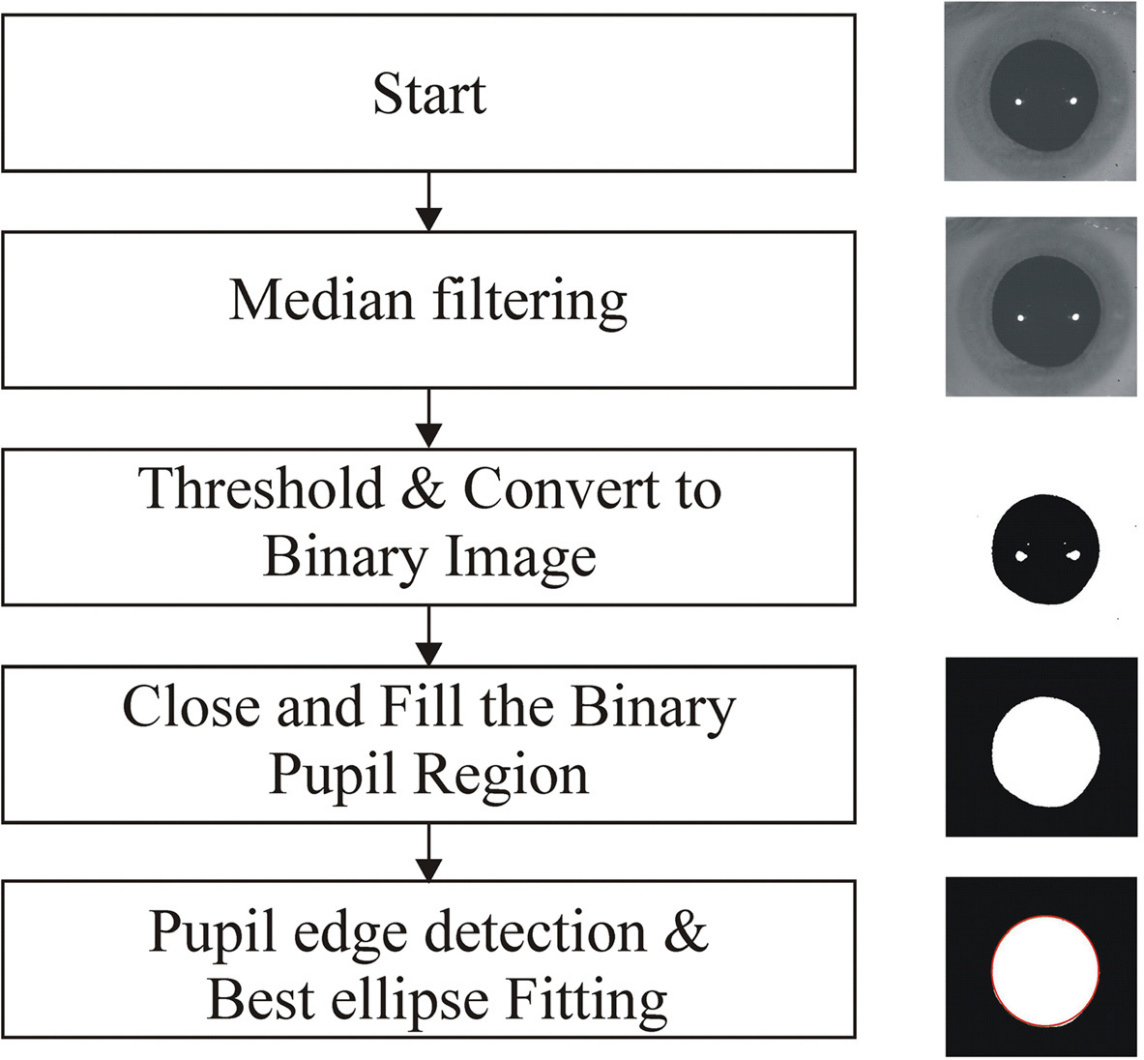
**Flowchart of the pupil image analysis algorithm.** The left panel shows the subsequent stages of the algorithm. The right panel shows how the analysis stage affects the pupil image.

The image processing module analyzes the recorded image sequence and estimates the measured pupil parameters frame by frame, individually for the left and the right eyes. We developed the algorithm using Vision Builder. It detects the pixels corresponding to the pupil image by considering the differences in intensity level, reflections, and the amount of ambient noise. It uses these pixels to calculate the pupil’s shape parameters. The flowchart of the algorithm is shown in Figure 4.

We smooth the image using median filtering (5 × 5 size) and remove any noise caused by changes in ambient lighting conditions. We use a thresholding procedure to remove potential artefacts (e.g., reflections) that arise during acquisition. The procedure calculates the average image intensity value from the image histogram, which it uses as a threshold to convert the image to a binary image. Because the images are often acquired under the same lighting conditions, the threshold is very similar for all images. Next, the identified region of interest (i.e., pupillary region) is closed and filled. When the pupil is isolated, we can detect the object by identifying the boundary coordinates of the pupil. The coordinates are used as input to fit the best ellipse using the least square criterion method. We then estimate the pupil parameters, that is, the coordinates of the pupil center, orientation, and minor and major axes of the equivalent ellipse. The obtained pupil dynamic waveforms (the time-varying parameters of the ellipse) are stored separately for the left and the right eye for further analysis. The eye blinking artefacts are removed by the averaging procedure.

## The light stimulator module

The light stimulator module is composed of two LED sources, one for the left eye (LED_OS) and one for the right eye (LED_OD). Figure 3 shows the stimulator attachment. It is a tube 45 mm in length and 10 mm in diameter. A lens with a focal length of 40 mm is located on one side (the closest to the eye). The LED is on the opposite side. A moving cross that can serve as the point of fixation is placed between the lens and the LED. A beam of stimuli light falls onto an eye in the Maxwellian projection (i.e., to the pupil center, with a light beam spot smaller than the minimum pupil diameter). This stimulates a retinal area with a diameter of approximately 20 mm. Each LED source can interchangeably be a white, red, green, or blue LED diode. The peak wavelengths of the three-color LEDs are 470 nm, 520 nm, and 625 nm with 5-nm half-height bandwidths. The output of each LED is computer controlled by a microprocessor using the pulse-width modulation technique. The light stimuli parameters can be adjusted by a system operator using the control panel. The adjustable parameters are: luminance level (from 1 to 100 cd/m^2^) and pattern (e.g., a pseudorandom signal, a single pulse, periodic signals (sinusoidal, triangular, and rectangular), or a positive/negative ramp).

## The controller module

**Figure 5:**
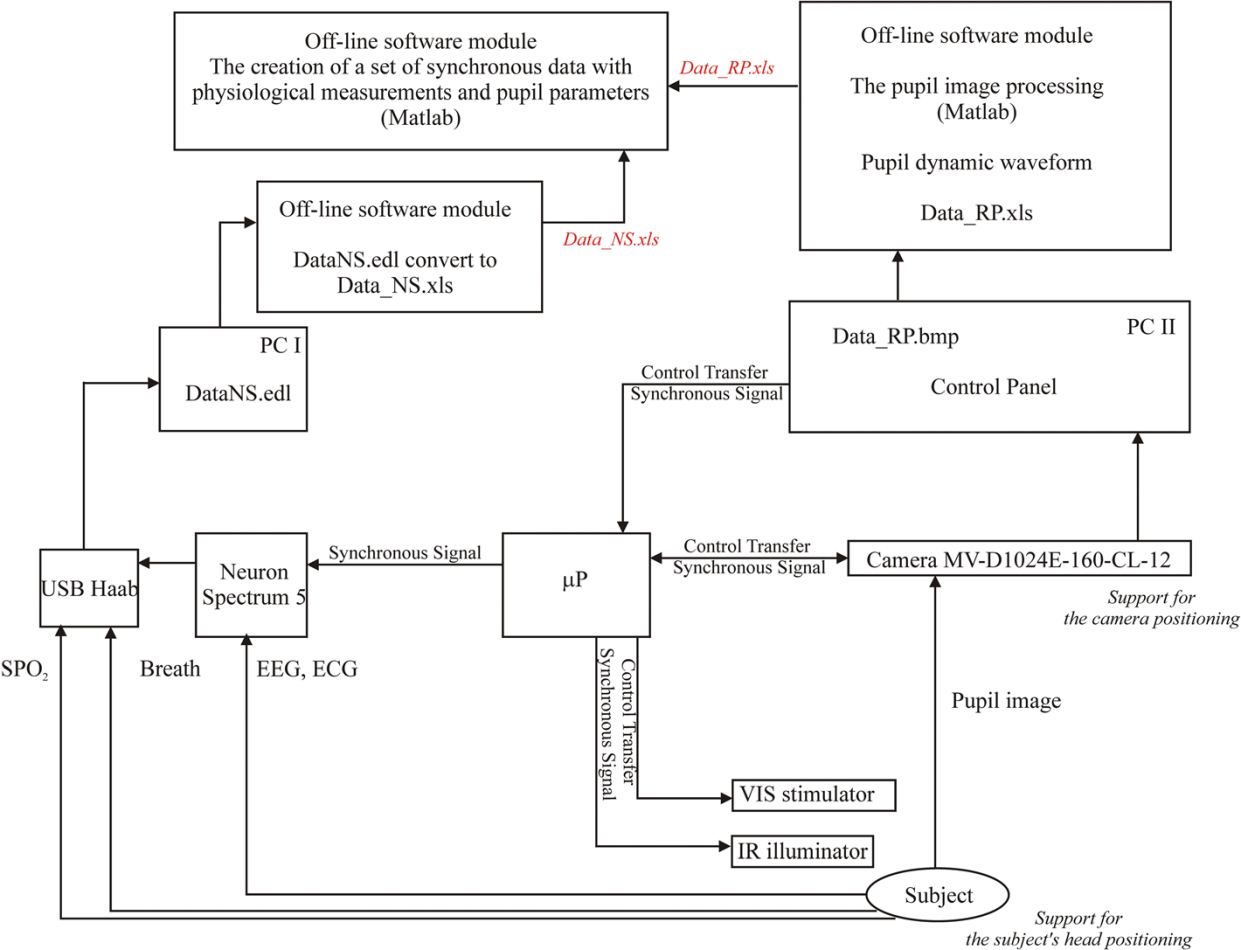
**Schematic diagram for synchronous operation of the pupillometer with the Neuron Spectrum.** The pupillometer is synchronised with the Neuron Spectrum by using a synchronous signal. Signals recorded in both systems have different formats, so they are analyzed offline.

**Figure 6:**
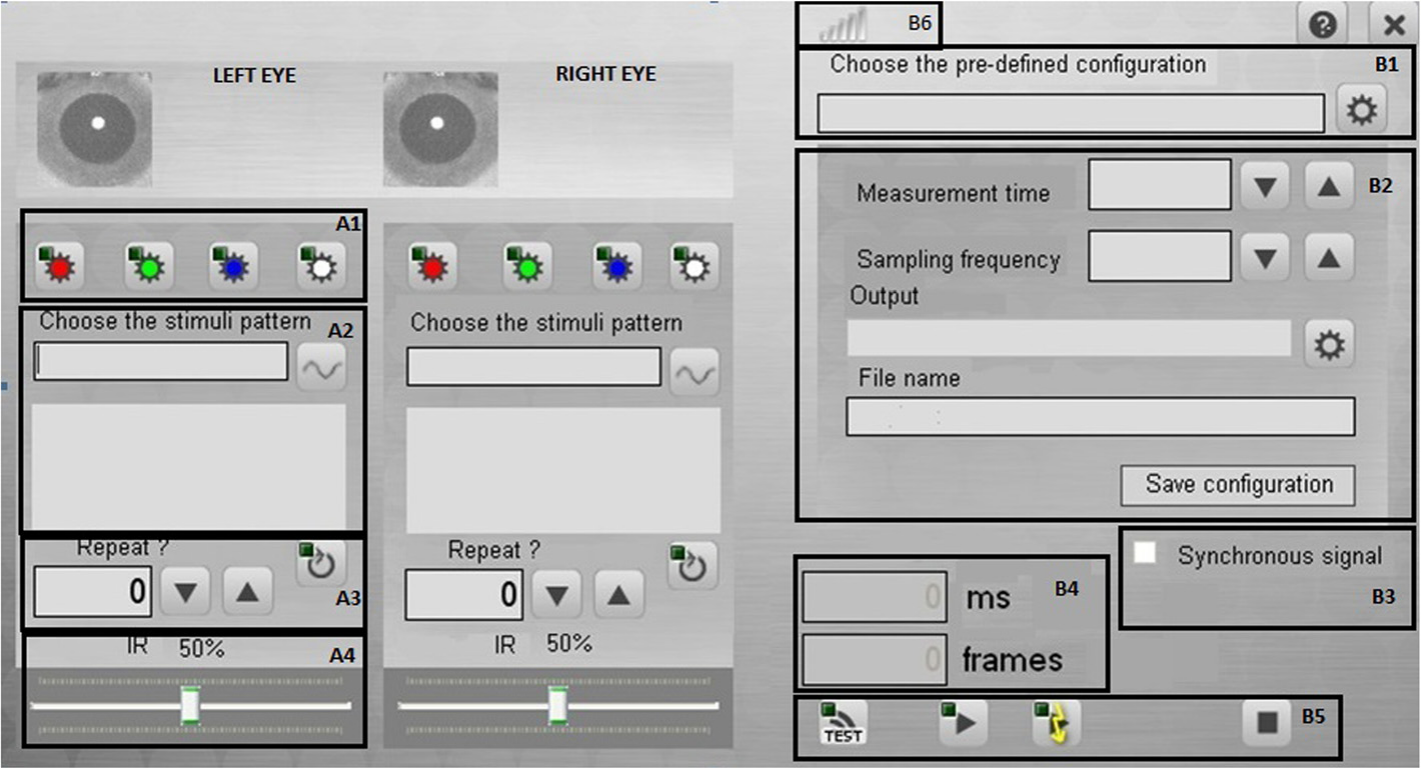
**Control panel.** The left part of the control panel sets the visual stimulation parameters (independently for the left and right eyes). The right part of the control panel configures the recording process.

The controller module is based on the ATXMega128A1 microprocessor, which we mainly chose because of its internal memory size and USB power supply (which is enough to power both the driver and connected LEDs). We developed the control software using C#. Additionally, the module has a synchronous output and can operate as a master with other bio-measurement systems. The microprocessor uses the level-controlled trigger mode of the camera, where the exposure time is defined by the pulse width of the external trigger signal. The length of the frame rate is defined as the distance between the successive rising edges of the synchronous signal. Additionally, the rising edge of the trigger signal is equivalent to sending the control pulse for light stimuli. Figure 5 shows the schema when the pupillometer is working synchronously with the Neuron Spectrum system. This system is now routinely used in our laboratory. A similar configuration can be used to connect the pupillometer to other bio-measurement systems with a trigger input.An operator controls the recording process and the stimuli parameters using a specially prepared control panel, which is shown in Figure 6.The control panel functions can be divided into two parts, A and B in Figure 6. Part. A sets the wavelength of the visual stimulation (A1), the loading pattern stimuli (A2), the flicker mode (A3), the start point of the stimulation relative to the beginning of the measurement (A3), and the IR light intensity (100% corresponds to 10 mW) (A4). These parameters are independently chosen for the left and the right eyes.

Part B sets the pre-defined recording configuration (B1), the recording configuration (B2), the synchronous signal (B3), and the current recording parameters (B4). Part B also controls the start/end of the test and measurements (B5), and verifies that the controller is correctly connected (B6).

The measurement is performed in scotopic conditions and is performed after a 5-min period where the eye adapts to darkness.

## System validation

To validate the system, we used three different sets of phantoms: a circle, an ellipse where the major axis is greater than the minor axis, and an ellipse where the major axis is less than the minor axis. We prepared two versions of each phantom, the first was a black image on a white background and the second was a black image with a white circle simulating a hypothetical reflection in the pupil caused by the IR LED. Each image was stored as a grayscale bitmap with 1024 × 1024 pixels, drawn using a commercial graphics editing program (Corel). The phantoms had similar dimensions to a real human pupil. The six black circle images were printed out using a high-level laser printer. The diameters of the printed images were accurately measured using a microscope with an accuracy greater than 0.01 mm. The diameters were 3.01, 3.96, 4.90, 5.83, 6.78, and 7.70 mm.

The aim of the first procedure was to test the analysis software. The results are summarized in Table 1.

**Table 1:** Results of the analysis software tests

	**Phantom**		**Ellipse fitting**	
	**Major axis [pixels]**	**Minor axis [pixels]**	**Major axis [pixels]**	**Minor axis [pixels]**
Circle phanton	119	119	118	118
	160	160	159	159
	201	201	200	200
	242	242	241	241
	283	283	282	282
	321	321	320	320
Ellipse phantom (major axis < minor axis)	119	160	118	159
	160	201	159	200
	201	242	200	241
	242	283	241	282
	283	321	282	320
Ellipse phantom (major axis > minor axis)	160	119	159	118
	201	160	200	159
	242	201	241	200
	283	242	282	241
	321	283	320	282

The algorithm provided correct results for the whole set of images. The analysis software results were less than 1% different to the measured sizes.

The second step evaluated the system resolution, and determined the accuracy and repeatability of the system. During our experiments, each phantom was placed on a tripod in a location that corresponded to the eye position in a real-time measurement. For each phantom, the 100-image sequence was recorded five times under comparable measurement conditions. We determined the phantom size in pixels for each picture. The mean and standard deviation (SD) values of our results are summarized in Table 2.

**Table 2:** Mean values and SD of measurements for each phantom

		**Phantom size after Ellipse fitting**											
**Phantom size**		**Major axis [pixels]**						**Minor axis [pixels]**					
[mm]	[pixels]	No 1	No 2	No 3	No 4	No 5	Mean	No 1	No 2	No 3	No 4	No 5	Mean
3.01	271	264 ± 20	272 ± 5	280 ± 9	275 ± 12	268 ± 24	273 ± 8	270 ± 3	272 ± 3	280 ± 16	275 ± 15	280 ± 12	275 ± 6
3,96	356	349 ± 20	362 ± 5	363 ± 13	357 ± 3	350 ± 8	356 ± 7	341 ± 33	362 ± 15	365 ± 8	352 ± 7	345 ± 28	354 ± 12
4,90	441	441 ± 8	452 ± 5	465 ± 30	450 ± 7	460 ± 28	452 ± 12	446 ± 8	462 ± 27	448 ± 3	452 ± 5	436 ± 29	450 ± 13
5,83	525	546 ± 12	537 ± 8	547 ± 13	542 ± 3	540 ± 4	542 ± 5	549 ± 21	535 ± 5	545 ± 3	539 ± 2	531 ± 16	541 ± 9
6,78	610	595 ± 25	619 ± 7	608 ± 3	599 ± 28	614 ± 5	608 ± 12	585 ± 35	617 ± 38	601 ± 4	595 ± 15	607 ± 7	602 ± 16
7,70	693	680 ± 5	693 ± 10	677 ± 22	691 ± 7	685 ± 2	685 ± 8	674 ± 20	694 ± 28	681 ± 5	684 ± 9	686 ± 8	685 ± 10

The linear resolution of the system was calculated using the ratio of the phantom size in millimeters to the phantom size in pixels. The ratio values were approximately 0.01 mm/pixel for all phantoms, so the linear resolution of the proposed system is estimated to be better than 0.02 mm. The accuracy of the timer is in the order of 0.2 μs, so we can neglect the errors in the time measurements.

We defined the system accuracy as the difference between the measured and true values. It was below 0.5% for all the considered phantoms. The standard deviation of the measurements was used as a measure of repeatability, and it was below 4% for all the considered phantoms.

**Figure 7:**
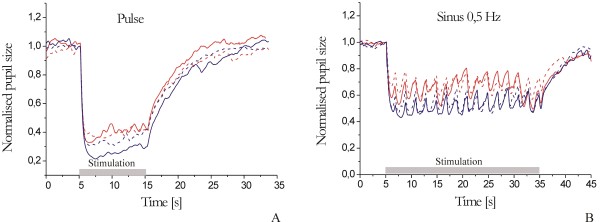
Pupil responses to chromatic light at a single 10-s pulse stimulus (A) and a flicker 0.5-Hz stimulus (B).

We performed several experiments to demonstrate the measurement possibilities of the system. Five healthy young subjects (24–37 years old) participated. All the subjects voluntarily participated in this experiment and agreed to the experimental procedure before it commenced. Some examples of the pupil recordings are presented below.Figure 7 presents the pupil light reflex (PLR) to chromatic light stimuli (red light shown using red lines and blue light shown using blue lines) at a single 10-s pulse (A), and a sinusoidal flicker of 0.5 Hz (B). The solid line is the response of the left eye and the dotted line is the response of the right eye.

**Figure 8:**
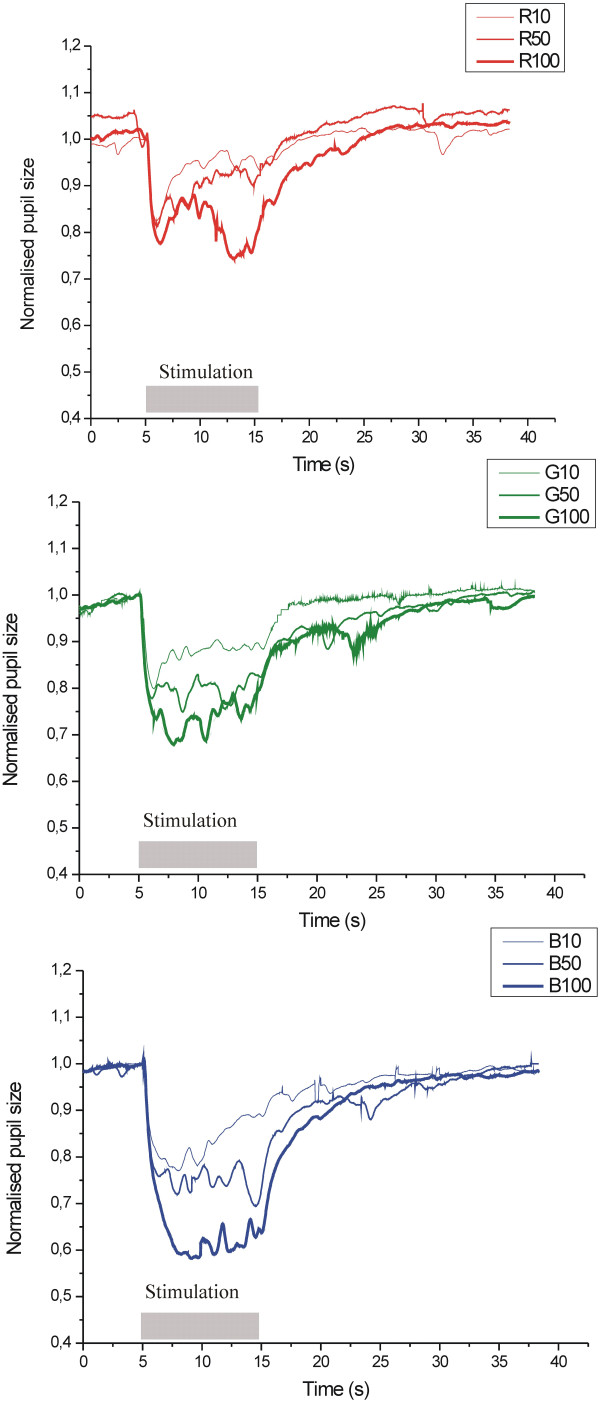
**Pupil light response to chromatic light stimuli at different luminance levels (10 cd/m**^**2**^**, 50 cd/m**^**2**^**, 100 cd/m**^**2**^**), for red (A), green (G), and blue (B) lights.**

**Figure 9:**
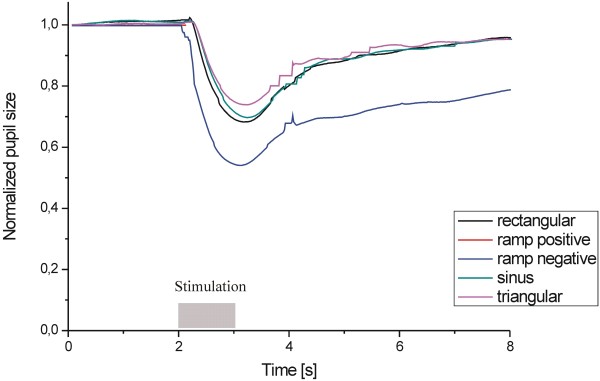
Pupil light response to white light for five different stimulus patterns.

**Figure 10:**
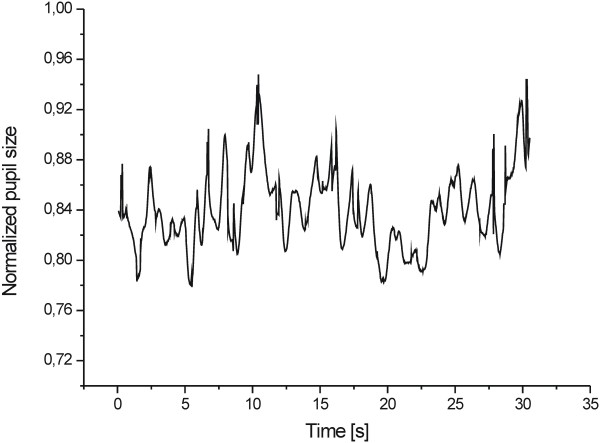
Spontaneous pupil fluctuations at a high luminance level.

Figure 8 shows the PLR to chromatic light stimuli at a different luminance levels (10 cd/m^2^, 50 cd/m^2^, and 100 cd/m^2^) for red (A), green (B), and blue (B) lights (the results are averaged for both eyes). Figure 9 shows the pupil light response (averaged for both eyes) to white light for five different stimulus patterns (triangle, rectangle, sine, positive ramp, and negative ramp). Figure 10 presents the spontaneous pupil fluctuation at a high luminance level.

## Discussion

In this article, we propose a binocular pupillometry system and measurement method. This system is currently used in our laboratory. It is a complete and low-budget system that can record comprehensive pupillometry measurements at high resolutions. The high precision parameters (75 Hz, 0.02 mm) for the binocular registration are better than those in the commercial and laboratory systems previously mentioned, which have nominal resolution ranges between 25 and 60 Hz and between 0.1 and 0.05 mm.

Our novel measurement method can monitor the right and left eyes using a single camera with no overlap between the pupil images. Our method requires precise synchronization of the IR lighting and camera work, which is done by the control module.

The advantage of the proposed measurement method is that we can use a small number of optical components (creating the optical path) and only one camera. This directly reduces the cost of the system, when compared with systems that use two cameras.

Although the mutual geometry of the optical components and camera is not very complicated, we require a high precision when designing the mechanical fixings and constructing the system. We prepared calibration phantoms that reflected the actual geometry of pupils, and used their size and mutual distance to adjust the system. Each fixing element has a motion system that allows for precise placement of the fixed elements. The more accurate this process is, the easier it is to calibrate the system with respect to individual subjects. This significantly reduces the biological noise level, especially with regard to eye blinks, and eye and head movements. Transversal head movements are limited by the head rest. Small transversal movements of the head do not substantially affect the accuracy. Likewise, small longitudinal head and eye movements do not substantially affect the image size of the pupil, because we use a telecentric lens to project the pupil image onto the camera. The fixation point stabilizes an accommodative pupillary reflex. The forehead height control system and joystick allow us to quickly and easily adjust the measuring unit relative to the subject’s head.

The proposed algorithm for pupil image analysis is not very complicated, when compared with previous methods (see Background). It consists of: median filtering, image thresholding, closing and filling the binary pupil region, detecting the pupil edge, and determining the pupil parameters modeled as an ellipse using a least-square criterion. Our simulations have confirmed that the best-ellipse approximation gives the correct results, especially for pupils with shapes that deviate from a circle. In the future, we will conduct a detailed analysis of other fitting algorithms.

The initial laboratory tests confirmed that the new system is a precise device (system accuracy greater than 0.5% and repeatability greater than 4%).

Our tests demonstrated the measurement possibilities of this system. We can record spontaneous pupil fluctuations and pupil light reflexes induced by light stimuli, independently controlling the parameters for each eye using a computer. In the proposed system, we can generate four classes of pupil response, the response of each eye when the right or left eye is stimulated, when both eyes are stimulated simultaneously, or when they are alternately simulated. Additionally, each eye can be stimulated using the same or different types of stimuli.

Our experiments conducted on a subject confirmed that the operator does not require much experience, because the system is relatively simple to use. It is also important that the system is convenient to the subjects. The prototype is mounted on the base of a slit lamp, which can be considered as a limitation. However, the system is constructed relative to the eyes, which means that it could be converted into a head-mounted system. We have not done this yet, because of the prohibitive size and weight of the available camera.

A distinguishing feature of our system is that it can cooperate with other bio-measurement systems with trigger inputs. We implemented this with systems that were available in our laboratory, i.e., a Neuron Spectrum system (41-channel multifunctional digital EEG system for neurophysiological studies) and a Biopac system (complete system for life science research and education). The proposed system can also cooperate with a new self-made laboratory version of the ophthalmic tonometer with a silicon micro-machined force sensor [23] for measuring intraocular pressure and ocular pulse.

Synchronous recordings of pupil reactivity and other physiological signals allows us to study correlations and coherence. This possibility significantly expands the research applications of pupillometry. Eventually, such a system could be an important step towards a pupillometer-based diagnostics system.

## Competing interests

The authors declare no competing interest with regard to this invited review.

## Authors’ contributions

WN developed the set-up of the hardware and software for the pupillometry system. AZ and ESP performed the pupillometric tests (phantoms and subjects) and worked as system operators. MMH recruited the subjects and designed the measurement protocols. WN drafted the manuscript, in consultation with the other authors. All authors read, reviewed, and approved the final manuscript.
